# Diagnosis of chronic rhinosinusitis in patients with cystic fibrosis: correlation between anamnesis, nasal endoscopy and computed tomography

**DOI:** 10.1016/S1808-8694(15)31236-2

**Published:** 2015-10-20

**Authors:** Letícia Boari, Ney Penteado de Castro

**Affiliations:** Master degree (Otorhinolaryngology); Ph.D. in Otorhinolaryngology, Joint Professor, Department of Otorhinolaryngology, F.C.M.S.C.S.P. Faculdade de Ciências Médicas da Santa Casa de São Paulo

**Keywords:** cystic fibrosis, sinusitis/diagnosis, endoscopy, paranasal sinus, clinical history, computed tomography

## Abstract

The sinonasal involvement is one of the most common manifestations in cystic fibrosis. Data show a high incidence of chronic rhinosinusitis in these patients. Although it has been found radiographic opacification of the sinus in more than 90% of cases, few are symptomatic. So that, it is difficult to recognize nasossinusal disease in patients with cystic fibrosis. Questionnaire, nasal endoscopy and CT-scan are very important methods in this approach.

**Aim:**

To evaluate the diagnosis of chronic rhinosinusitis in patients with cystic fibrosis by anamnesis, nasal endoscopy and CT-scan and compare those results.

**Study Design:**

Clinical prospective.

**Material and Method:**

Evaluation of 34 patients - older than 6 years and with a confirmed diagnoses of cystic fibrosis - by anamnesis (questionnaire), nasal endoscopy (score Lund-Kennedy) and CT-scan (score Lund-Mackay).

**Results:**

chronic rhinosinusitis was confirmed in: 20,58% of cases by the questionnaire, 73,52% of the cases by the nasal endoscopy and in 93,54% of the cases by the CT-scan. The results showed significant differences. The correlation between nasal endoscopy score (Lund-Kennedy score) and CT-scan score (Lund-Mackay score) was statistically significant.

**Conclusion:**

The diagnosis of chronic rhinosinusitis was statistically different between the three methods. It was higher in imaging analysis and lower in questionnaire. The nasal endoscopy is an excellent method to evaluate nasossinusal disease in cystic fibrosis

## INTRODUCTION

Cystic fibrosis is a disease that should be part of our ENT knowledge because it has many manifestations in our area of work, especially those related with nasosinusal impairment.

It is a recessive autosomal genetic disease, characterized by a set of signs and symptoms resulting from impairment of exocrine glands and respiratory, digestive and reproductive tracts. It is more incident in the Caucasian population, affecting 1:2,000 live births in many countries^1^.

In the end of the 80's, the gene of cystic fibrosis was identified, located in chromosome^7^. This gene is responsible for codifying the regulating protein of cystic fibrosis transmembrane conductance (CFTR)^2^. In the respiratory tract, the impairment of epithelial hydroelectrolytic transport by CFTR dysfunction causes affection to the viscoelastic properties of mucus. It provides greater susceptibility to respiratory infections such as pneumonia, bronchitis, bronchiectasia and rhinosinusitis. Progressive pulmonary disease determines respiratory failure, which is still the main cause of death by cystic fibrosis^3^. It is believed that nasosinusal impairment may exacerbate the pulmonary affection, given that it serves as a bacterial reservoir^4^. Thus, the appropriate importance of the approach to the paranasal sinuses affections in these patients is quite evident.

In the literature, there is the description of high incidence of chronic rhinosinusitis in patients with cystic fibrosis^5^^,^^6^. There is radiological impairment in almost all paranasal sinuses in more than 90% of the patients aged over 8 months of age 7. However, these findings are not always followed by symptoms^8^. Some authors sustain the hypothesis that patients get adapted to their life condition, underestimating the symptoms^6^^,^^7^. There is controversy about the valuation of clinical assessment findings, nasofibroscopy and imaging exams in the investigation of chronic rhinosinusitis, a fact that hinders the diagnosis of the disease.

This fact has given rise to the interest of studying how Otorhinolaryngologists can contribute to the diagnosis of chronic rhinosinusitis in patients with cystic fibrosis, aiming at improving quality of life. The purpose of the present study was to assess the diagnosis of chronic rhinosinusitis in patients with cystic fibrosis using anamnesis (questionnaire), nasofibroscopy and computed tomography (CT scan) of the paranasal sinuses and by comparing their findings.

## MATERIAL AND METHOD

The study was carried out jointly by the Departments of Otorhinolaryngology and Pediatrics of the institution, and the project and the informed consent term were approved by the Research Ethics Committee under protocol number 346/04. All patients were informed about the study, agreed to participate and signed the Free Informed Consent Term. Data collection was prospective and occurred during November 2004 to February 2005. To comprise the study sample, we selected subjects that were part of the outpatient unit of Pediatric Pneumology, who met the inclusion criteria and did not fit the exclusion criteria listed below:

– Inclusion criteria: diagnosis of cystic fibrosis confirmed by the sweating test (≥ 60mEq/L)^10^ in subjects aged over 6 years. Children under 6 years were not included in the sample because they would require general anesthesia to undergo CT scan.
– Exclusion criteria: subjects that did not cooperate in the performance of nasofibroscopy and/or CT scan of paranasal sinuses; history of nasosinusal surgery or facial trauma, and consequent modification of anatomical parameters of the assessment, acute infectious of the upper airways, which could have hindered or masked the diagnosis of chronic rhinosinusitis. Persistent symptoms for up to 4 weeks were considered as acute manifestations^9^.

The sample was comprised by 34 patients and the age range varied from 6 to 22 years, mean age of 12.26 years (standard deviation of ± 4.29). Out of the total of studied patients, 15 were male. As to ethnics, 85.3% were Caucasian and the remaining were Black; no Asian subjects were included. Out of 34 patients, 21 presented genotyping with identification of mutating allele ΔF508 in 57.14% of the cases.

In the sample, 30 patients were using low-dose Azythromycin during assessment.

The selected patients were assessed with anamnesis, comprising a directed questionnaire, nasofibroscopy and paranasal sinuses CT scan. The questionnaire comprised major and minor symptoms of chronic rhinosinusitis - nasal obstruction, nasal secretion/post-nasal drainage (major), facial pain, headache and cough (minor). Responses were classified into absent (0) or present (1) and included the approximate duration of each symptom. We considered positive clinical history for chronic rhinosinusitis if the subject referred two or more major symptoms or one major and two or more minor symptoms that had lasted ≥ 12 weeks^11^.

Nasofibroscopy was performed on the same day using flexible optical fiber of 3.2mm, Machida brand, coupled to a microcamera and videocassette system. The exams were recorded in VHS tapes, and they were all performed and analyzed by the same examiner (LB). We used endoscopic staging proposed by Lund-Kennedy^12^ to assess the following parameters: nasal mucosa edema, presence of secretion and presence of polyps. For each one of them, we scored 0 to 2, as shown in Chart 1. This assessment was performed bilaterally, with the total points corresponding to the sum of values obtained in both sides. Thus, the score ranged from 0–12.

**Chart 1:** Lund-Kennedy score of endoscopic assessment.

Characteristics	Nasal cavity	
	right	left
Polyp (0,1,2)		
Edema (0,1,2)		
Secretion (0,1,2)		
total		

**Note:** Polyp: 0 - absent; 1 - limited to the middle meatus; 2 - extending to the nasal cavity

Mucosa edema: 0 - absent; 1 - mild/moderate edema; 2 - polypoid degeneration

Secretion: 0 absent; 1 - hyaline; 2 - thick and/or mucopurulent

The endoscopic result was considered positive for chronic rhinosinusitis if Lund-Kennedy score was higher than 2.

CT scan of paranasal sinuses was scheduled in the Department of Radiology of the institution in a maximum period of 3 weeks after the questionnaire and nasofibroscopy, provided that there were no modifications to nasosinusal symptoms. We performed sections at coronal and axial plans with continuous sections of 2.0 and 3.0mm thickness. CT scans were assessed by examiner LB, based on Lund-Mackay^14^ scale. Each paranasal sinus was graded from 0 to 2 depending on the level of opacification (Chart 2). The total score was 0–24 points, and the highest value corresponded to greater severity of the disease. Only pneumatized sinuses were scored. To enable the comparison of results, we corrected the original score from 0–24. Thus, we multiplied the value obtained by 24 (total number of pneumatized paranasal sinuses)^15^.

**Chart 2:** Lund-Mackay score of CT scan.

paranasal sinuses	Right	Left
Maxillary (0,1,2)		
Anterior Ethmoid (0,1,2)		
Posterior Ethmoid (0,1,2)		
Sphenoid (0,1,2)		
Frontal (0,1,2)		
Ostiomeatal Complex (0,2)*		
Total		

Note: 0 - without abnormalities; 1 - partial opacification; 2 - total opacification

* 0 - no obstruction; 2 - obstructed

For the statistical analysis of the collected data, in addition to the descriptive analysis, we used the following techniques: confidence interval, equal test of two proportions, comparison of univariate means (ANOVA). The level of significance in all tests was 0.05 (or 5%).

## RESULTS

The main referred complaints were: cough (88.2%); headache or facial pain (38.2%) and nasal obstruction (29.1%). Rhinorrhea was reported in only 4 patients. Considering the symptoms, only 20.58% of the 34 patients were diagnosed as having chronic rhinosinusitis.

**Graph 1:**
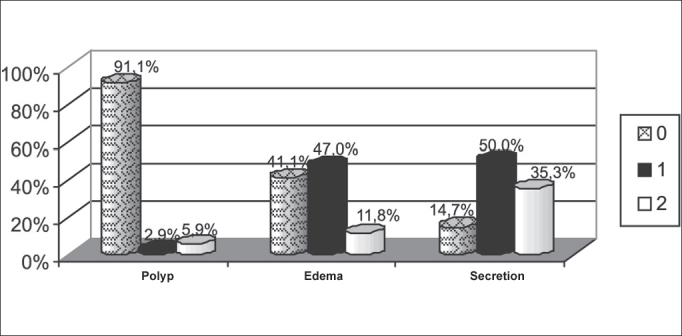
Percentage demonstration of endoscopic assessment using Lund-Mackay score in the sample. - Note: Polyp: 0 - absent; 1 - limited to the middle meatus; 2 - extending to the nasal cavity. Edema: 0 - absent; 1 - mild/moderate; 2 - severe/polypoid degeneration. Secretion: 0 absent; 1 - hyaline; 2 - purulent and thick.

The result of nasofibroscopy based on Lund-Kennedy score was demonstrated in Graph 1, according to the endoscopic criteria for the diagnosis of chronic rhinosinusitis in the sample, 73.52% of the patients presented the disease at the time of the assessment.

CT scan was performed in 31 subjects, because 3 of them did not come for the exam. Imaging staging based on Lund-Mackay presented average of 13.3, ranging from 1 to 24 points.

The most affected paranasal sinus was the maxillary sinus, with 91.9% of opacification − 45.1% of complete opacification and 46.8% of partial opacification. Next, in decreasing order, there was affection to anterior ethmoid (83.9%), frontal (70%), sphenoid (66.7%) and posterior ethmoid (54.8%). Considering the CT scan parameters for diagnosis of chronic rhinosinusitis, we detected that 93.54% of the 31 patients presented the disease.

**Graph 2:**
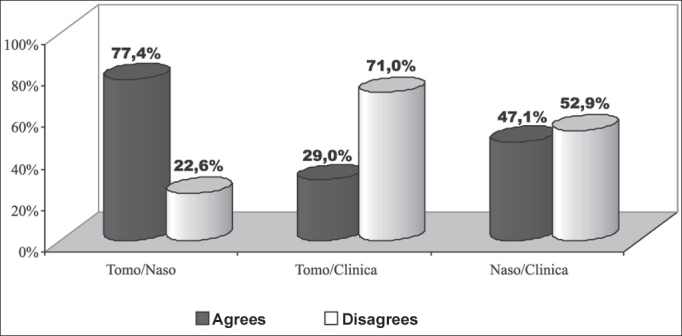
Percentage demonstration of agreement of results of assessments in relation to diagnosis of chronic rhinosinusitis of the sample: CT scan, nasofibroscopy and questionnaire.

Upon comparing the three diagnostic methods two by two, we noticed that they presented results with statistically significant differences (p < 0.001) and CT scan was the exam that presented the higher level of positive results (93.54%) and the questionnaire presented the highest percentage of negative responses (79.42%). CT scan and nasofibroscopy presented higher level of agreement, whereas CT scan and the questionnaire had the highest level of disagreement (Graph 2).

**Figure 1:**
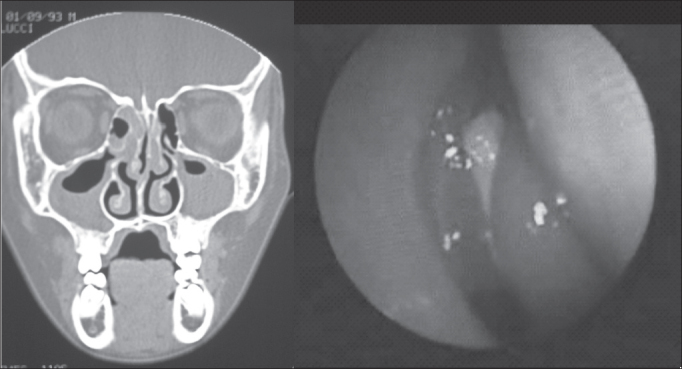
Example (case 12) of the agreement between results of assessments. CT scan and nasofibroscopy with positive diagnosis of chronic rhinosinusitis.

**Figure 2:**
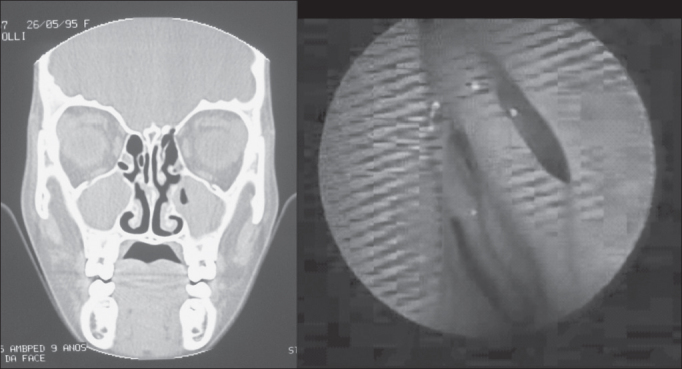
Example (case 5) of disagreement between the results of assessments. CT scan with positive diagnosis and nasofibroscopy with negative diagnosis of chronic rhinosinusitis.

Figure 1, Figure 2 are examples of agreement and disagreement, respectively, between the results of nasofibroscopy and CT scan for the diagnosis of chronic rhinosinusitis in the sample. In both examples, the assessment of symptoms by the questionnaire was negative for nasosinusal disease.

## DISCUSSION

It is a consensus among researchers in the area that nasosinusal disease is practically universal in cystic fibrosis^6^. As a result, the participation of Otorhinolaryngologists in the follow-up of these patients is becoming more and more expressive. In the past, the professionals used to focus the attention on relieving symptoms, with palliative management. Currently, it is the Otorhinolaryngologist' responsibility to maintain the upper airways in good conditions, contributing to prevent infectious presentations^5^. It is essential that specialists decide what is the right time and how to intervene. To that end, he/she needs enough data to correctly make the diagnosis of nasosinusal disease and to identify how much it is interfering in the patients' quality of life. This difficult decision has motivated the investigation of the topic.

In our sample, we tried to have the most homogeneous sample possible to minimize biases. Mean age of selected patients was within the age range that had the most nasosinusal symptoms in mucoviscidosis, which, according to Ramsey and Richardson (1992)^7^, takes place at 5 to 14 years.

The diagnostic criterion of chronic rhinosinusitis should be rigorous to prevent false-positive results. Nosological diagnosis should be made by the association of symptoms, signs shown in nasofibroscopy and paranasal sinuses CT scan^11^. These were the criteria accepted for our methodology. Nasofibroscopy and CT scan staging used in the study were Lund-Kennedy and Lund-Mackay scores, which are universally accepted, facilitating the comparison with other studies^12^^,^^14^^,^^15^.

The sample of this study presented distribution by gender, race and genotyping equivalent to that demonstrated in other studies^1^^,^^3^. Out of 34 studied subjects, 30 had used prophylactic Azythromycin for pulmonary inflammatory condition. Studies showed that this class of antibiotics in low doses and for prolonged periods of time acted mainly as an immunomodulator of the inflammatory response, and there was not enough plasma concentration to have a bactericide action16., 17., 18..

Except for cough, the occurrence of rhinosinusitis symptoms reported in the study was low. We should carefully analyze the complaint of cough, because it may be related only with the pulmonary presentation. These data are similar to those found by Shapiro et al. (1982)^19^, King (1990)^8^ and Cuyler (1992)^20^.

Differently, some studies reported high incidence of nasosinusal complaints. Some hypotheses were developed to explain this fact. The studies by Brihaye et al. (1997)^21^ and Coste et al. (1995)^22^ have possibly presented disagreeing results owing to the different age range studied and the higher incidence of nasal polyposis in their samples.

Gentile and Isaacson (1996)^23^, in a retrospective study reported nasal obstruction in 62% of the cases and headache in 31% of the 16 charts they analyzed. In addition to the fact the sample was small and there were incomplete data in the medical charts, the studied group had been operated on, which means that they probably had more symptoms. Rowe-Jones and Mackay (1996)^24^ studied 46 patients with mucoviscidosis that were operated on for chronic rhinosinusitis refractory to clinical treatment. Authors described their sample as representative of the most advanced level of chronic rhinosinusitis in the population with cystic fibrosis. Thus, more symptomatology would have been expected in such study.

Therefore, the study reinforced the low occurrence of nasosinusal symptoms in cystic fibrosis when the assessment of all patients is made and not only in those scheduled to be operated on. The valuation only of symptoms for the diagnosis of chronic rhinosinusitis is insufficient, demanding objective exams. Moss and King (1995)^25^ justified this fact by the following factors: patients and/or responsible people prioritize attention to other clinical manifestations of the disease that are more severe, underestimating the nasosinusal complaints; there is adaptation of the symptoms, reducing the discomfort caused by them, there is lack of knowledge about the nasosinusal disease and it may interfere in quality of life and progression of pulmonary condition. Normally, these should be some of the main reasons for low occurrence of nasosinusal symptoms.

In the sample, 73.5% of the patients presented predictive signals of chronic rhinosinusitis by nasofibroscopy. Three of the 34 cases presented polyps, 35.3% presented mucopurulent secretion and 58.8% presented mucosa edema. These results are similar to those described by Coste et al. (1995)^22^ and Brihaye et al. (1997)^21^ except for the polyps that were detected by the authors with higher incidence, respectively in 43.7% and 33% of the subjects.

The incidence of nasal polyps is variable in the literature from 6.7% to 48%; this variability may be attributed to different age ranges that comprise the studied samples and the different assessment methods25., 26., 27., 28..

It is known that CT scan assessment is abnormal in practically 100% of the patients with cystic fibrosis^25^. In the present study, the imaging diagnosis was positive in 93.45% of the 31 cases. Out of the total of 24-point score of Lund-Mackay, the mean score was 13.2 (± 6.64). Similar results were described by Yung et al. (2002)^29^ in the routine assessment of 23 patients. They reported that only 2 cases presented almost normal CT aspect (score < 4). Differently, many studies performed CT scan only in severe nasosinusal cases, before surgery. Rowe-Jones and Mackay (1996)^24^ studied a selected sample of pre-surgical patients by chronic rhinosinusitis and polyposis. CT scan according to Lund-Mackay score before the surgery revealed an average score of 9.5 (± 2.1) of the maximum score of 12 (unilateral), considering only the worst side. To each patient, there was an average of 81% of paranasal sinuses opacification. However, they reported that the severity of the nasosinusal disease demonstrated in the imaging exam does not seem to be directly related with the surgical findings. It is probably due to the CT scan limitation to distinguish between the purulent secretion or mucous or the edema mucosa.

Considering individually each of the assessments for the diagnosis of chronic rhinosinusitis, we observed that there was statistically significant difference between them. The positive diagnosis of the disease was predominantly observed in CT scan and it was negative for assessment of symptoms (questionnaire). Moreover, considering the results of agreement and disagreement relative to the disease, the study showed that CT scan and nasofibroscopy presented higher percentage of agreeing results whereas CT scan and the questionnaire presented the highest proportion of disagreeing results. These differences were statistically significant.

The possible explanations to justify these results are the following: the diagnosis of chronic rhinosinusitis determined only by the symptomatology is underestimated because the patient does not value the nasosinusal condition, as suggested by other authors^7^^,^^25^. Moreover, the fact that the patients in the study were submitted to preventive treatment of pulmonary infections with Azythromycin could have contributed to minimize the upper airway symptoms. As to CT scan, there is probably underestimation of the diagnosis of nasosinusal disease. Some studies show that in some cases there is lack of correlation between surgical findings and CT scans 24 and even postoperatively, and CT scan should not be used as a parameter to control the disease, given that it is probably affected with paranasal sinuses opacification, which does not correspond to signs and symptoms^20^.

Nasofibroscopy revealed chronic rhinosinusitis in 73.5% of the cases and in our opinion it is the most sensitive assessment to this affection. It should be periodically performed in patients with cystic fibrosis, even when there is no suspicion of rhinosinusitis in the clinical assessment, because it may evidence relevant nasosinusal abnormalities.

In view of all the aspects previously presented, the contribution that Otorhinolaryngologists may give to the follow up of cystic fibrosis patients is remarkable. The multidisciplinary approach, with constant exchange of information and experience is the ground to reach the main objective, that is, patients' wellbeing. In past decades, there have been many significant achievements in quality of life and survival of patients with cystic fibrosis. We do hope it is a continuous stimulus for us to overcome our limitations and always focus on the common goal of cure.

## CONCLUSION

The data analysis in our study led to the following conclusions:

1. The positive diagnosis of the disease is predominantly observed by imaging exams - CT scan.
2. The negative diagnosis is predominantly observed by the assessment of symptoms - anamnesis.
3. There is statistically significant difference between the assessment tools for the diagnosis of chronic rhinosinusitis.
4. Nasofibroscopy has expressively contributed to the assessment of chronic rhinosinusitis in patients with cystic fibrosis, reliably characterizing all nasosinusal conditions.
